# China's campaign against rare diseases

**DOI:** 10.1093/nsr/nwac015

**Published:** 2022-01-29

**Authors:** Weijie Zhao

In 2018, *National Science Review* interviewed Prof. David R. Liu of the Broad Institute, who pioneered CRISPR-based genome-editing technologies [1]. As soon as this interview was posted on China's social media, a father of a Duchenne muscular dystrophy (DMD) patient contacted us, hoping Liu's innovation could save his son. The DMD patients usually lose the ability to stand and walk by the age of 12, and many do not survive beyond 20 years old. Similarly to rare-disease families throughout the world, rare-disease patients in China are desperately in search of possible therapy. Since 2015, attention on rare diseases has been mounting in China, with emerging new policies and programs. Compared with their counterparts in the USA and Europe, rare-disease patients in China are facing many more challenges in receiving diagnosis and treatment. On 18–19 December 2021, the 2021 China Conference on Rare Diseases was held online. Hundreds of physicians, researchers, policy-makers, patients and medical-industry representatives gathered to discuss multiple aspects of rare diseases in China.

## PROGRESS SINCE 2015

The definition of rare disease differs among various countries. In the USA, according to the Rare Diseases Act of 2002, it is defined as diseases affecting <200 000 people in the USA or with a patient ratio of <1/1500. But in China, there is no commonly accepted definition.

Throughout the world, there are 7000–10 000 types of rare diseases known, affecting 350 million patients, among whom 20–54 million are Chinese. Although the total patient number is high, patients are rare for a given disease and the phenotypes can be highly diverse. Thus, rare diseases are very difficult to diagnose and treat—many physicians are not familiar with the diseases and ∼95% of them have no effective therapies.

The legislation of the Orphan Drug Act (ODA) of 1983 marked the beginning of the US government’s supporting research and development (R&D) of rare-disease drugs (orphan drugs). The number of orphan drugs approved by the US Food and Drug Administration rose from 38 to 1031 during the period of 1983–2021 [2]. Similar ODAs became available in Australia in 1990, Japan in 1993 and the European Union in 2000.

China has not yet passed any legislation on rare diseases or orphan drugs, but multiple polices and strategies have been implemented since 2015. As introduced by Director of Peking Union Medical College Hospital (PUMCH) Shuyang Zhang and other experts at the conference, the first National Expert Committee on Diagnosis, Treatment and Insurance of Rare Diseases was formed in 2015; the List of Rare Diseases (First Batch) including 121 diseases was released in 2018 and the second batch is coming soon. During 2016–2018, National Natural Science Foundation of China invested 57.2 million yuan (∼9 million US dollars) into basic research on rare diseases. The National Rare Disease Sample Base has been built and a three-level prevention and control network for rare diseases comprising 324 hospitals nationwide has been established, providing much better medical information and treatment for these patients. Moreover, two national shared databases on rare diseases have been established: the National Rare Disease Direct Reporting System has collected 540 000 cases of rare diseases from 291 institutions and the National Rare Diseases Registry System has included >68 000 cases of 171 rare diseases [3].

Nevertheless, China's campaign against rare diseases is still at the beginning phase. It involves science and technology advances, as well as economic and societal issues.

## TO DIAGNOSE RARE DISEASES

About 80% of rare diseases are genetic in origin. Since the beginning of the 2010s, second-generation sequencing technology has triggered a revolution of the diagnosis of rare diseases. Today, whole-exome sequencing, together with mitochondrial DNA sequencing, proteomic analysis and other technologies, enable physicians to make definite diagnosis of many rare diseases much easier than before—as long as such sequencing tests are requested by the physicians.

But sequencing is not a magic solution. The first problem is that not all pathogenic gene variations are known. Research articles discovering new variations appear daily and the Online Mendelian Inheritance in Man database is updated daily. But the clinical sequencing companies need a much longer time (≥3 months) to update their testing chips and their database. Furthermore, the reported pathogenic gene variations may be false positives. Professor Yanling Yang from Peking University First Hospital provided an example on the conference: a mutation of phenylalanine hydroxylase (PAH) on the 53rd amino acid was first detected in phenylketonuria (PKU) patients; it had long been considered to be pathogenic and treatment was derived. But after decades of clinical practice, it was found that this mutation also exists in the genome of some healthy people and the mutation itself is not responsible for PKU [4]. It is difficult to identify the real positive variations from the massive amount of data and it is important for the physicians to combine the clinical phenotypes and the sequencing results in order to achieve a reliable diagnosis.

According to the 2020 China Rare Disease Comprehensive Social Research, among the 38 000 surveyed medical workers, 32% reported that they had never diagnosed or treated rare diseases and 66% admitted that they were not familiar with rare diseases. In such circumstances, despite the availability of sequencing tests and other diagnosis methods, patients are still unlikely to be quickly identified. Rare-disease education for medical students and medical workers is an urgent issue. In recent years, Chinese textbooks for rare diseases have appeared and rare-disease courses are offered to both medical students and clinical workers throughout China.

For the diagnosis of rare diseases, another key word is multi-disciplinary team (MDT). Under the fine subdivision of modern medical systems, specialized physicians are usually familiar with limited categories of diseases and are unlikely to make the correct diagnosis by themselves when encountering the rare and confusing symptoms of rare-disease patients. Thus, MDT diagnosis involving doctors from different disciplines is needed. In PUMCH, a top comprehensive hospital in Beijing, MDT workshops on rare diseases have been held weekly since 2019 and have benefitted >127 patients. Dozens of senior physicians from multiple disciplines, including hospital Director Shuyang Zhang, attend the workshops to analyse the symptoms, make diagnoses and design specific treatment plans in a patient-centered manner.

Other comprehensive hospitals are also implementing similar MDT programs, but for most patients who live outside big cities, such an opportunity remains rare. To alleviate this problem, 324 hospitals across China formed a rare-disease network that enables online MDT workshops among different hospitals. Moreover, PUMCH is sharing its MDT workshops online with >100 hospitals throughout China so that doctors in these hospitals can learn about rare-disease cases. With this national hospital network, some patients could be definitely diagnosed with rare diseases within several weeks rather than years, as in the past.

**Figure:**
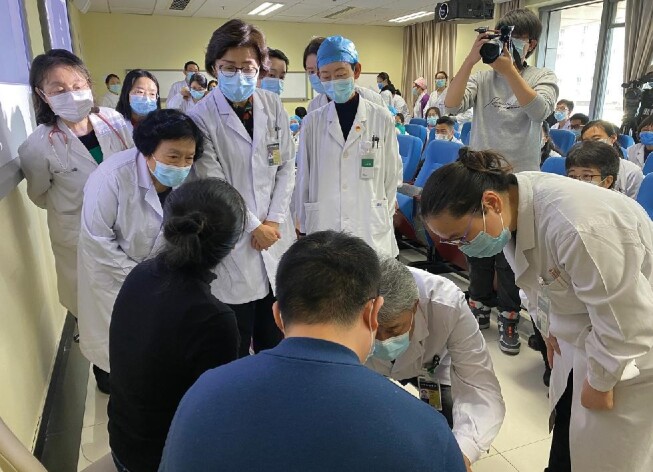
Rare-disease MDT workshop at PUMCH. (*Courtesy of PUMCH*)

## TREATMENTS FOR RARE DISEASES

Once diagnosed, the bad news is that for ∼95% of rare diseases, no drug or therapy is available. Pharmaceutical industry has largely stayed away from orphan-drug R&D because of the lack of a financial incentive for diseases with a very low number of patients. For the patients, there is no drug or, even if there is a drug, it is too expensive. When commercial initiative is low, government policies should help. The ODAs in several countries have greatly encouraged orphan-drug R&D by pharma companies but there is still a rather long way to go.

Rare diseases, especially the genetic ones, are attractive targets for new biotechnologies such as cell therapy and CRISPR-based gene therapy. Many pioneering scientists, including Feng Zhang, David Liu and Nobel laureates Jennifer Doudna and Emmanuelle Charpentier, have entered this field and founded their own biotech companies. However, there is a specific challenge for orphan-drug R&D—the difficulty in recruiting enough patients for randomized double-blind clinical trials. So among the orphan-drug trials, there are many single-arm trials without control groups. Moreover, real-world studies, which are often retrospective studies of real-world cases, are also emphasized in rare-disease research, especially for the discovery of the efficiency of an approved drug for a new disease.

The R&D capacity of Chinese pharmaceutical companies still lags far behind those in the Western countries. Encouraged by the priority review and data-protection polices in recent years, some local innovative pharmaceutical companies have started orphan-drug R&D and several biotech companies focusing on rare diseases have been established. Hundreds of rare-disease clinical trials have also begun in China. For example, there are at least four international multicenter clinical trials for DMD ongoing in China, involving two drugs: Pamrevlumab and Ataluren [5]. However, with all this progress, among the 121 diseases listed in China's List of Rare Diseases (First Batch)—among the 7000 known types—only 74 have available drugs; among these 74 diseases, drugs for 16 were developed in other countries and are not available in China.

Because of the rarity, drugs for rare diseases are unlikely to be regularly reserved in a hospital so most patients have to wait for their drugs to be transported from another hospital, another city or another country. For the 16 rare diseases whose drugs have not been approved for clinical use in China, Hainan Boao Lecheng International Medical Tourism Pilot Zone offers an exceptional chance. Physicians in this Pilot Zone can apply for one-time import of drugs or medical equipment urgently needed in clinic. Successful one-time import examples include Osilodrostat for Cushing's Syndrome and Naglazyme for Mucopolysaccharidosis Type VI.

In spite of these opportunities, there is another hurdle for most rare-disease families—the extremely high price of the orphan drugs. More than 50% of rare-disease patients are children and about one-third of them die before the age of 5. Many parents are willing to pay anything they have to save their children's life but the price of available drugs, on the other hand, is beyond reach for many families. According to Luwen Shi,

Director of International Research Center for Medicinal Administration, Peking University, patients in China can obtain financial support from four channels: (i) the national basic health insurance, which has included >40 drugs for 25 rare diseases, most of which are low-price drugs; (ii) the national supplementary medical insurance that includes some other drugs; (iii) special funds and charity foundations that cooperate with some hospitals to support rare-disease patients; (iv) commercial insurance—many Chinese cities have begun to offer policy-supported commercial insurance with a relatively low price. However, this support is limited and cannot cover all the expenses for rare-disease families. More government and social support from all channels is urgently needed.

## SOCIAL SUPPORT

‘To cure sometimes, to relieve often, to comfort always.’ This famous epitaph describes the life of American physician Edward Livingston Trudeau (1848–1915), who had been a tuberculosis patient himself. He established a sanitarium for tuberculosis patients at the Saranac Lake and had relieved and comforted many patients throughout his life. In this conference, Yi Dai from PUMCH compared today's rare diseases to tuberculosis and other infective diseases during Trudeau's time—they may be difficult or even impossible to cure, but physicians and families can provide relief and comfort for the patients by creating comfortable living conditions. Bin Chen is a DMD patient born in 1993. With the support of his family and society, he has not only lived longer than most DMD patients, but also became a PhD student of psychology in Tsinghua University.

**Figure:**
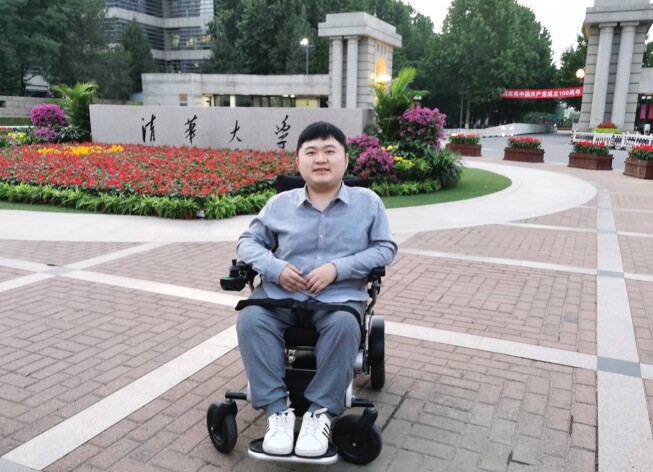
DMD patient Bin Chen is a PhD candidate of psychology in Tsinghua University. *(Courtesy of Bin Chen)*

The China Alliance for Rare Diseases (CARD) was established in 2018. With PUMCH, China Pharmaceutical Innovation and Research Development Association, Chinese Hospital Association and Chinese Research Hospital Association as the leading members, CARD unites hospitals, universities, research institutions, companies and patient organizations for promoting basic research, policy study, clinical treatment and public education on rare diseases. According to a CARD-led survey in 2019, there are at least 74 patient organizations in China. These organizations connect hundreds or thousands of patients together and offer them multiple resources and emotional support. Notably, many of these organizations are established and led by rare-disease patients or their families. As examples, the Chinese Organization for Rare Disorders is initiated and led by pseudoachondroplasia patient Rufang Huang and the Chinese Organization for Scleroderma is led by scleroderma patient Ai Zheng. Many patients and their families are trying their best to help themselves and their companions.

It is a great misfortune for the families afflicted with rare diseases. Chinese society has begun to see them and to take action to help. There is hope, and there will be cures.
